# Integrated Bayesian models of learning and decision making for saccadic eye movements^☆^

**DOI:** 10.1016/j.neunet.2008.08.007

**Published:** 2008-11

**Authors:** Kay H. Brodersen, Will D. Penny, Lee M. Harrison, Jean Daunizeau, Christian C. Ruff, Emrah Duzel, Karl J. Friston, Klaas E. Stephan

**Affiliations:** a Wellcome Trust Centre for Neuroimaging, Institute of Neurology, University College London, 12 Queen Square, London WC1N 3BG, UK; b Centre for Functional Magnetic Resonance Imaging of the Brain (FMRIB), John Radcliffe Hospital, University of Oxford, Oxford OX3 9DU, UK; c Institute of Cognitive Neuroscience, University College London, 17 Queen Square, London WC1N 3AR, UK; d Branco-Weiss-Laboratory, Institute for Empirical Research in Economics, University of Zurich, Switzerland

**Keywords:** Saccades, Decision making, Reaction time, Bayesian learning, Model comparison

## Abstract

The neurophysiology of eye movements has been studied extensively, and several computational models have been proposed for decision-making processes that underlie the generation of eye movements towards a visual stimulus in a situation of uncertainty. One class of models, known as linear rise-to-threshold models, provides an economical, yet broadly applicable, explanation for the observed variability in the latency between the onset of a peripheral visual target and the saccade towards it. So far, however, these models do not account for the dynamics of learning across a sequence of stimuli, and they do not apply to situations in which subjects are exposed to events with conditional probabilities. In this methodological paper, we extend the class of linear rise-to-threshold models to address these limitations. Specifically, we reformulate previous models in terms of a generative, hierarchical model, by combining two separate sub-models that account for the interplay between learning of target locations across trials and the decision-making process within trials. We derive a maximum-likelihood scheme for parameter estimation as well as model comparison on the basis of log likelihood ratios. The utility of the integrated model is demonstrated by applying it to empirical saccade data acquired from three healthy subjects. Model comparison is used (i) to show that eye movements do not only reflect marginal but also conditional probabilities of target locations, and (ii) to reveal subject-specific learning profiles over trials. These individual learning profiles are sufficiently distinct that test samples can be successfully mapped onto the correct subject by a naïve Bayes classifier. Altogether, our approach extends the class of linear rise-to-threshold models of saccadic decision making, overcomes some of their previous limitations, and enables statistical inference both about learning of target locations across trials and the decision-making process within trials.

## 1 Introduction

In order to survive in a competitive, dynamic environment, animals must be able to integrate past experience with sensory evidence to infer the current state of the world and execute a behavioural response. Marked progress in our understanding of the neural basis of decision making has been achieved by focusing on sensory-driven decisions, such as the simple question of where to look next. Studying decision making in sensorimotor systems like the oculomotor system has the advantage that one can exploit a large body of neuroanatomical and neurophysiological knowledge that has been accumulated over the past decades. It seems conceivable that studying the neuronal mechanisms of visual-saccadic decision making could provide us with a blueprint of how the brain implements other sensorimotor decisions, or even deliver “a model for understanding decision making in general” (Glimcher, 2003).

The decision processes that underlie rapid eye movements towards a target have been studied in a variety of experimental paradigms. One seminal series of studies is based on the *random dot-motion* task designed by Newsome and colleagues (Newsome & Pare, 1988). In an initial fixed-duration version of this task, monkeys were trained to discriminate the motion direction of a set of moving dots with varying degrees of coherence, and indicate the perceived motion by a leftward or rightward saccade (Newsome, 1997; Newsome, Britten, & Movshon, 1989; Newsome, Britten, Salzman, & Movshon, 1990; Salzman, Britten, & Newsome, 1990). Subsequently, Shadlen, Britten, Newsome, and Movshon (1996) suggested a computational explanation of the neuronal mechanisms producing the resulting saccade and provided experimental verification of its key assumptions (Gold & Shadlen, 2000; Kim & Shadlen, 1999; Shadlen et al., 1996; Shadlen & Newsome, 2001). In particular, they identified a gradual rise of spiking activity in the lateral intraparietal (LIP) area integrating motion direction-specific signals from the middle temporal (MT) area (Shadlen & Newsome, 1996, 2001).

Based on a reaction time version of the same task (Roitman & Shadlen, 2002), Shadlen and colleagues advanced the hypothesis that rising activity before a saccade, which had also been observed in the frontal eye fields (FEF), represented the ratio of the log likelihoods that the two possible eye movements would be executed (Gold & Shadlen, 2000, 2001). Based on their decision-theoretic analysis, they suggested that log likelihood ratios might be used as “a natural currency for trading off sensory information, prior probability and expected value to form a perceptual decision” (Gold & Shadlen, 2001).

Another key series of studies was carried out by Hanes, Schall, and colleagues, who investigated an *oddball* task (as well as the *countermanding* paradigm; Hanes and Carpenter (1999)) to study how neural signals in the FEFs would finally trigger the initiation of saccades (Hanes & Schall, 1996; Hanes, Thompson, & Schall, 1995; Schall & Thompson, 1999; Thompson, Bichot, & Schall, 1997; Thompson, Hanes, Bichot, & Schall, 1996). In their oddball task, monkeys were trained to indicate, by an eye movement, the location of the oddball within a circular arrangement of visual stimuli around a central fixation dot. They showed that FEF activity was consistent with psychophysical models about oddball reaction time tasks (Luce, 1986; Ratcliff, 1978; Sternberg, 1969a, 1969b). Specifically, their findings supported the notion that the saccadic decision would be made as soon as gradually increasing neural activity in the FEFs had crossed a biophysical threshold (Hanes, Patterson, & Schall, 1998; Schall & Thompson, 1999).

Motivated by the question of why saccadic latencies displayed large variance in all of the above tasks, an even simpler reaction time paradigm was investigated by Carpenter and colleagues (Carpenter & Williams, 1995; Reddi & Carpenter, 2000). In their *saccade-to-target* reaction time task, human subjects were asked to shift their gaze from a central fixation stimulus to an eccentric target as soon as it appeared on the screen. The critical manipulation was to vary the uncertainty about where the target would appear (Basso & Wurtz, 1997, 1998). It was found that saccade latencies became shorter with increasing prior probability of the corresponding target location. Specifically, response speed was found to be proportional to the log prior probability of target location (Basso & Wurtz, 1997, 1998; Carpenter & Williams, 1995).

The behavioural and electrophysiological findings from all three paradigms described above are consistent with the notion of a saccade being elicited once some gradually rising neuronal activity crosses a biophysical threshold. This idea has been formalized in terms of various mechanisms known as *rise-to-threshold* accumulator models. These models aim to provide a computational abstraction of a biophysically conceivable mechanism that explains saccade latencies and their variability across trials (for reviews see Glimcher (2001, 2003), Gold and Shadlen (2001), Platt (2002), Ratcliff and Smith (2004), Schall (2001, 2003), Smith and Ratcliff (2004) and Usher and McClelland (2001)).

In the context of saccadic decision making with a fixed set of potential target locations, rise-to-threshold models assume that subjects maintain a set of hypotheses each of which corresponds to one such location (Carpenter & Williams, 1995; Gold & Shadlen, 2002; McMillen & Holmes, 2006; Shadlen & Gold, 2004). As the stimulus appears, a measure of evidence for each of these hypotheses is continuously refined, implemented as a competition between alternative decision signals in the brain. At any given point in post-stimulus time, these decision signals might, for example, represent the posterior probabilities of the target hypotheses, as derived from the subject’s prior (Basso & Wurtz, 1997, 1998; Platt & Glimcher, 1999) and the sensory evidence (i.e., the likelihood of the data) collected up to that point in time (Carpenter, 2004; Carpenter & Williams, 1995). As soon as one such signal reaches a preset threshold, a saccade is elicited towards the corresponding target. Depending on the way in which information is assumed to be accumulated over time, two specific types of rise-to-threshold model are often distinguished: random-walk models and linear rise-to-threshold models.

*Random-walk* or *diffusion* models are fundamentally based on a sequential probability ratio test that is being carried out continually (Ratcliff, 1978; Ratcliff & Rouder, 1998; Ratcliff & Smith, 2004; Ratcliff, Zandt, & McKoon, 1999; Wald, 1945). In these models, each new incoming piece of sensory evidence either increases or decreases a single decision variable until it has drifted beyond a threshold associated with the saccadic movement towards a particular target. The decision variable represents the relative evidence for the two alternatives (Ratcliff & Rouder, 1998). However, in the case of a simple saccade-to-target task in a high-contrast setting with highly salient targets, it has been questioned whether a random-walk process for target detection provides a sufficient explanation for the large variability in latencies (Carpenter, 2004; Carpenter & Reddi, 2001; Reddi, 2001).

In *linear* rise-to-threshold models, randomness is introduced as trial-by-trial changes in the otherwise constant rate of rise of the decision signal. This notion has been formalized by Carpenter in a model termed ‘LATER’ (linear approach to threshold with ergodic rate; Carpenter and Williams (1995), Leach and Carpenter (2001), Reddi, Asrress, and Carpenter (2003)). Like other rise-to-threshold models, LATER proposes that a saccade towards a target is elicited as soon as a neural decision signal has reached a particular threshold. But unlike other rise-to-threshold models (e. g., Grice (1968) and Nazir and Jacobs (1991)), it assumes a fixed threshold and a linear increase whose rate is subject to variation *across* trials, yet fixed *within* a given trial (for a debate on the relationship between the two approaches see Carpenter and Reddi (2001), Ratcliff (2001), Usher and McClelland (2001)). The neurophysiological recordings by Schall and colleagues (Hanes & Schall, 1996; Schall & Thompson, 1999) are consistent with these key assumptions of the LATER model: they had observed that the threshold for saccade release seemed to be constant, whereas the slope of the rise in activity varied considerably across trials (see Fig. 2a).

In their experiments on the saccade-to-target task, Carpenter and colleagues found that the observed saccadic latency was a function of the log probability of the corresponding target location: the more likely the target location, the shorter the latency (Carpenter & Williams, 1995). LATER accounts for this relationship by assuming that the learned a priori target probabilities determine the baseline levels of the decision signals, but not their rates of rise (cf. biased choice theory by Luce (1963)). Carpenter and colleagues used LATER to produce remarkably accurate predictions of human latency distributions in the saccade-to-target task as well as variations of it (Asrress & Carpenter, 2001; Carpenter & Williams, 1995; Leach & Carpenter, 2001; Reddi et al., 2003; Reddi & Carpenter, 2000).

A strength of the LATER model is the straightforward interpretability of its parameters. LATER has thus been used to relate various features of observed latency distributions to the putative underlying neurophysiological process (Anderson, 2008; Asrress & Carpenter, 2001; Carpenter & McDonald, 2007; Kurata & Aizawa, 2004; Leach & Carpenter, 2001; Loon, Hooge, Berg, & den, 2002; Madelain, Champrenaut, & Chauvin, 2007; Reddi et al., 2003; Sinha, Brown, & Carpenter, 2006). Furthermore, various extensions have been proposed, such as arrangements of multiple LATER units in parallel (Carpenter, 2004; Robinson, 1973), mixture models (Nakahara, Nakamura, & Hikosaka, 2006), or the assumption that both the rate of rise and the baseline level of the decision signal are trial-by-trial random variables (Nakahara et al., 2006).

However, the simplicity of this model limits its applicability in three ways. First, linear rise-to-threshold models like LATER have only been applied to saccade-to-target situations in which no learning took place: in previous studies, prior probabilities of target location were always fixed in a given experimental session, and subjects were initially given extensive training until their performance levelled off. During learning, by contrast, the baseline levels of the decision signals are expected to *change* across trials. Even though the notion of variable baseline levels has been discussed before (Glimcher, 2001; Nakahara et al., 2006), no specific model has been put forward how they might evolve dynamically depending on the history of previous trials. Second, LATER only accounts for simple marginal probabilities, where the probability distribution of target locations is described by a single vector of probabilities. It does not account for higher-order contingencies, that is, situations in which the target location probability depends on the target location during the previous trial. Third, within the class of linear rise-to-threshold models, no generative model has been proposed so far that would allow for statistical inference about parameter estimates and for model comparison (e.g., with regard to the type of learning that occurs across trials).

In this study, we propose a more general linear rise-to-threshold model for visual-saccadic decision making that overcomes the restrictions outlined above. First, we explicitly model how subjects’ priors are systematically altered by the sequence of stimuli observed so far. This approach makes it possible to investigate how learning dynamically shapes decision making about saccades. Second, our model is able to account for different forms of learning which can be evaluated by model comparison. In particular, this allows us to investigate whether subjects’ behaviour is not only driven by *marginal* but also by *conditional* probabilities. Third, based on computational considerations, we propose a specific parameterization of the model. This enables parameter estimation within a maximum likelihood scheme and the subsequent construction of a classifier that can be used to distinguish subjects with different learning profiles.

## 2 Methods

### 2.1 Task

For the present study, subjects were engaged in a sequential reaction time task (SRTT) during which they had to elicit saccades towards a given target in quick succession. The predictability of the target location was modified between blocks to induce varying forms of learning. The degree to which subjects learned the underlying contingency of a particular block was measured by the latencies of their saccades, that is, the time between stimulus onset and the beginning of the saccade towards the stimulus.

The specific setup adopted in this study was based on the saccade-to-target task proposed by Carpenter and Williams (1995). Subjects placed their heads on a chinrest in front of a computer screen in a dark, soundproof booth. At the beginning of a trial, they focused on a red fixation dot (hue 0^∘^, luminance 0.5) at the centre of a black screen. After a random waiting period between 500 and 1500 ms, a second red dot, the target, appeared on the screen, either located at 15^∘^ to the left or to the right. Since the original fixation dot remained visible, this design represented an overlap task rather than a gap task (alternative types of waiting-period probability distribution are examined in Oswal, Ogden, and Carpenter (2007)). Subjects were asked to foveate the target as quickly as possible, but not at the cost of errors. After another 700 ms, both dots disappeared, and the screen remained blank for an inter-trial interval of 500 ms.

Based on this design, Carpenter and Williams (1995) investigated the effects of fixed state probabilities for the two target locations on saccadic reaction times. For example, prior to the actual experiment, subjects were trained extensively on a sequence of trials during which the target appeared on the left-hand side with a probability of 70%, and on the right-hand side with a probability of 30%.

**Fig. 1:**
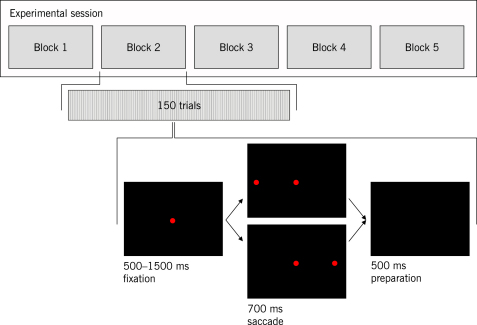
Experimental design. A complete session consists of 5 blocks, each of which contains 150 trials generated from the same block-specific transition matrix. All matrices shown in the main text were used to generate samples in each session. A trial consists of three consecutive stages: a fixation screen (showing a central red fixation dot); a target screen (showing both the fixation dot and an additional leftward or rightward target dot); an inter-trial interval (showing a black screen until the beginning of the next trial).

In our study, we extended this experimental design in two ways (see Fig. 1 ). First, each block contained a comparatively small number of trials, and subjects were not trained on a particular setting before the beginning of data acquisition. In this way, data were acquired while learning was in progress. Experimental pilots showed that 150 trials allowed for the subjects’ performance to stabilize sufficiently. Second, in addition to modifying target probabilities across blocks, the probability *structure* underlying the sequence of target locations was varied.

In a *state-oriented block*, as in previous experiments, the sequence of target locations was generated according to fixed state probabilities. They were specified as a vector (p,1−p), 0≤p≤1, where p and 1−p denote the marginal probabilities of leftward and rightward targets, respectively.

In a *transition-oriented block*, the probability distribution of the target location of the current trial was conditional on the target location of the previous trial. Thus, given a sequence of past trials, the probability distribution of the target on the next trial depended on the last item of the sequence, and only on this one. A sequence with this property is known as a first-order Markov chain, and the change from one trial to the next as a transition. The probability that the next target location is j, given that the current target location is i, is given by $p_{i,j}$. Thus, the sequence of target locations was specified by the transition matrix of its underlying Markov chain, $P={\left[p_{i,j}\right]}_{1\leq i,j\leq 2}$, where $p_{i,1}$ and $p_{i,2}$ denote the probabilities of leftward and rightward targets, respectively, given that the target of the previous trial appeared at location i. The first target in the sequence was drawn from a uniform initial distribution π=(0.5,0.5); that is, the sequence of target locations was initialized randomly, either with a leftward or with a rightward target. The example in Table 1 shows a short sequence of trials generated from the transition matrix

$$P=\left[\begin{array}{cc} 0.7 & 0.3\\ 0.3 & 0.7 \end{array}\right]\text{.}$$

For each trial, the table shows the probability distribution vector from which the current target location is drawn. For the first trial, it is (0.5,0.5). In all subsequent trials, it is either the top or the bottom row of P, depending on whether the previous target location was ‘left’ (top row) or ‘right’ (bottom row). The example illustrates that, given a transition matrix with high diagonal probabilities (a ‘stable’ transition matrix), the target tends to stay where it was on the previous trial, and only occasionally switches to the other side.

Finally, in a *uniform block*, target locations occurred on the left-hand side and the right-hand side with equal chance, rendering the sequence of targets maximally unpredictable. This block structure served as a control condition in which no statistical learning across trials should take place.

In order to avoid drowsiness, which subjects in pilot experiments had displayed after 30 min of constant testing, a single experimental session was chosen to contain only 5 blocks. A break of 3 min between any two blocks was introduced to reduce the potential confound of learning effects carrying over from one block to another.

In order to allow for a unified formalism, all blocks were specified in terms of a transition matrix P. The blocks for each session were chosen according to the scheme in Box I .

Each session contained all five block types. Their order was randomized in each session, and the two alternative matrices underlying the state-oriented blocks were counterbalanced across subjects. In order to distinguish transition-oriented learning from simpler state-oriented learning, all transition-oriented blocks were designed in such a way that the states of the implied Markov chain, 1 and 2, had a uniform steady state distribution $\pi^{*}=\left(0.5,0.5\right)$ (see Papoulis (1991)). Hence, in transition-oriented blocks, targets would, on average, appear equally often on either side, and no state-oriented learning should take place.

Experimental data were collected from three healthy male right-handed authors of this article with normal vision aged between 23 and 40 years (KHB, KES, WDP; see Table 2 ). Eye movements were recorded at a sampling frequency of 120 Hz using an ASL 504 infrared remote optics eye tracker. Targets were presented on a 27 cm × 37 cm CRT screen at a viewing distance of 67 cm. Data acquisition and analysis were implemented using MATLAB, Cogent 2000, and ILAB (Gitelman, 2002).

Before extracting latencies from eye recordings, blinks were filtered by searching for invalid pupil size values. Pupil coordinates within a time window of 25 ms around the beginning and the end of a blink were removed. Saccades were then detected using a standard algorithm by Fischer, Biscaldi, and Otto (1993): in the raw recorded eye coordinates we looked for an initial pupil velocity of 250^∘^/s and searched the consecutive 100 ms time window for a saccade of at least 10^∘^ that resulted in a fixation of at least 100 ms. Any latencies below 10 ms or above 800 ms were interpreted as artifacts and removed, as were blocks with an overall recognition rate below 80%. Altogether, 15% of the recorded blocks were rejected, as were 4% of the trials from accepted blocks. For the remaining trials, we computed the latency between target onset and the beginning of the first detected saccade.

In order to reduce the variance of latencies, each subject took part in many sessions with an overall number of more than 20 000 trials.

### 2.2 Modelling

Various models have been proposed over the past two decades to explain the variability in the latencies between the appearance of a target and the initiation of an eye movement towards it. In one class of models, a decision signal is assumed to rise at a linear rate until reaching a fixed threshold. The release of a saccade is then modelled as the final outcome of this linear rise-to-threshold mechanism (Carpenter & Williams, 1995; Leach & Carpenter, 2001; Reddi et al., 2003). This type of model can be extended in two ways: (i) within an individual trial, the linear rise to threshold can be parameterized and turned into a generative model; (ii) across trials, the dynamics of alternative forms of learning can be integrated into the model.

The two levels can be formalized as hierarchically related *intra-trial* and *inter-trial* sub-models, respectively. They are described separately in the following sections. Put together, they predict saccade latencies on the basis of the sequence of target locations observed so far, as well as three model parameters.

#### 2.2.1 Intra-trial modelling

We propose a generative intra-trial model that extends previous models of the relation between prior expectations about target location and saccadic onset times. It describes a computational abstraction of putative neurophysiological processes between target onset and the release of a saccade.

**Fig. 2:**
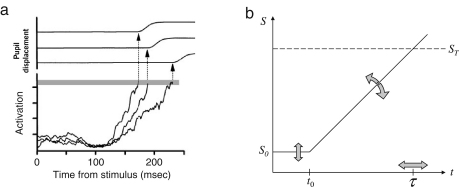
Neuronal responses of a decision process and translation into a computational model. (a) Neuronal responses prior to a saccade from three trials. In their experiment, Schall and Thompson (1999) trained rhesus monkeys to stare at a central fixation stimulus and, as soon as eight secondary targets appeared, to elicit a saccade towards the oddball. The targets were arranged radially around the central fixation stimulus, and the location of the oddball was random. The diagram shows the recorded activity of single movement-related neurons in the saccadic movement maps of the frontal eye fields (FEF). Trials were grouped into those with slow, medium and fast saccades. The three plots show the averaged activity within these groups of trials, in each trial taking the activity from that neuronal response field corresponding to the correct target location. The activity patterns show that there is a fairly constant biophysical threshold at which a saccade is irrevocably elicited (grey bar) whereas the rate at which the signals rise varies between the groups of saccades. (Reprinted, with permission, from the Annual Review of Neuroscience, Volume 22 (c) 1999 by Annual Reviews, www.annualreviews.org) (b) Translation into a computational model of the decision process for a single trial. The rising activation in the FEFs is modelled as a linearly rising decision signal S. It starts off at an initial level $S_{0}$ and rises at a variable rate until reaching threshold $S_{T}$ at time τ.

Each trial has two potential outcomes: a target appears either on the left-hand side or on the right-hand side of the screen. Within each trial, we model a subject’s belief in these two outcomes at any given point of time as distinct decision signals S that evolve linearly over time t until one of them hits a threshold $S_{T}$. At the beginning of a trial, both decision signals have specific initial values, with the signal of the ‘winning’ hypothesis defined to start at $S_{0}$. The initial level $S_{0}$ reflects the subject’s prior as provided by the inter-trial model described in Section 2.2.2. As the stimulus appears at $t=t_{0}$, the two signals increase or decrease, respectively, representing the changing belief in the two hypotheses. As soon as one of them hits threshold, a saccade is released towards the corresponding target location at time τ. The rate at which the decision signals rise or fall varies over trials, accounting for the large variance of the resulting latency distribution (see Fig. 2).

The decision process can be parameterized by modelling subjects as Bayesian observers who collect evidence about the true state of the world and, combined with their prior expectations, accept one of two competing hypotheses about it (Knill & Pouget, 2004). In this scheme, the initial level of the ‘winning’ decision signal, $S_{0}$, is associated with the subject’s prior, and the rate of rise is associated with the likelihood of the true hypothesis.

Evidence from several studies provides support for this parameterization. For instance, based on psychophysical experiments in humans, Carpenter and Williams (1995) plotted latencies on a reciprobit scale, in which normally distributed data approach a straight line. They found that a change in the marginal probabilities of the two target locations led to a reciprobit swivel. This change in slope is consistent with a change in the threshold height but not with a change in the mean rate of rise, which would cause a reciprobit shift (Sinha et al., 2006). Further support for the notion that priors determine the initial level of the decision signal rather than its rate of rise comes from neurophysiological experiments using a similar task to ours in monkeys (Basso & Wurtz, 1997, 1998; Ratcliff, Cherian, & Segraves, 2003). These studies identified neurons in the superior colliculus whose firing rates just before target onset reflected the target probability but not target salience. Altogether, these human and primate studies provide a robust foundation for the assumption that the subjective prior systematically influences the initial level of the decision signal, $S_{0}$, before it starts to rise until hitting threshold.

Let the two possible states of the world be denoted by $h_{i},\;i\in \left\{1,2\right\}$, corresponding to the target location being $x_{k}=i$, respectively, within the current trial k. The sensory evidence for the hypotheses $h_{i}$ is provided by time-continuous visual input. Assuming this supportive evidence to be processed in small, discrete timesteps, in a lossless fashion without any form of temporal filter (Ludwig, Gilchrist, McSorley, & Baddeley, 2005), the evidence at time t is referred to as $e_{t}$, and the accumulated evidence for one or another hypothesis up to time t is denoted by $e_{1..t}$. Writing $p\left(e_{t}\right)$ for the probability density of the piece of evidence at time t, we make two simple assumptions. First, it is assumed that $p\left(e_{2}|e_{1},h_{i}\right)=p\left(e_{2}|h_{i}\right)$, that is, $e_{1}$ and $e_{2}$ are conditionally independent. This means that $p\left(e_{t}|e_{1..t-1},h_{i}\right)=p\left(e_{t}|h_{i}\right)$. Second, since sensory stimuli $e≔e_{1}=\ldots =e_{t}$ are equal throughout the duration of the trial, the likelihood term $p\left(e|h_{i}\right)$ is taken to be constant. It follows that

(1) $$p\left(e_{1..t}|h_{i}\right)=p\left(e_{t}|e_{1..t-1},h_{i}\right)\times p\left(e_{t-1}|e_{1..t-2},h_{i}\right)\times \cdots \times p\left(e_{1}|h_{i}\right)=p\left(e_{t}|h_{i}\right)\times p\left(e_{t-1}|h_{i}\right)\times \cdots \times p\left(e_{1}|h_{i}\right)=p{\left(e|h_{i}\right)}^{t}\text{.}$$

Subjects are modelled as permanently testing a decision rule which determines whether they continue their observation—or accept one of the hypotheses. From a Bayesian learning perspective it is intuitive to consider, as a decision variable, the subjective posterior probability of each hypothesis, given the supporting evidence up to time t. In an iterative form, its dynamics can be written as

(2) $$P\left(h_{i}|e_{1..t}\right)=P\left(h_{i}|e_{t},e_{1..t-1}\right)=\frac{p\left(e_{t}|h_{i},e_{1..t-1}\right)\times P\left(h_{i}|e_{1..t-1}\right)}{p\left(e_{t}|e_{1..t-1}\right)}=\frac{p\left(e_{t}|h_{i}\right)\times P\left(h_{i}|e_{1..t-1}\right)}{p\left(e_{t}|e_{1..t-1}\right)}\text{,}$$

illustrating how the prior probability $P\left(h_{i}|e_{1..t-1}\right)$ is turned into a posterior probability $P\left(h_{i}|e_{1..t}\right)$ as new evidence $e_{t}$ is processed. Using Bayes’ theorem and Eq. (1), we obtain the closed form

(3) $$P\left(h_{i}|e_{1..t}\right)=\frac{p\left(e_{1..t}|h_{i}\right)\times P\left(h_{i}\right)}{\overset{2}{\underset{i=1}{\sum }}p\left(e_{1..t}|h_{i}\right)\times P\left(h_{i}\right)}=\frac{p{\left(e|h_{i}\right)}^{t}\times P\left(h_{i}\right)}{\overset{2}{\underset{i=1}{\sum }}p{\left(e|h_{i}\right)}^{t}\times P\left(h_{i}\right)}\text{,}$$

in which the assumption of discretized time is no longer necessary. However, this quantity does not rise linearly over time. Therefore, as an alternative decision variable that can be constructed in the case of two possible target locations, we consider the log posterior ratio. Using (2), its iterative form can be written as

(4) $$\ln\frac{P\left(h_{1}|e_{1..t}\right)}{P\left(h_{2}|e_{1..t}\right)}=\ln\frac{P\left(h_{1}|e_{1..t-1}\right)}{P\left(h_{2}|e_{1..t-1}\right)}+\ln\frac{p\left(e_{t}|h_{1}\right)}{p\left(e_{t}|h_{2}\right)}\text{.}$$

Using (3), the closed form is

(5) $$\ln\frac{P\left(h_{1}|e_{1..t}\right)}{P\left(h_{2}|e_{1..t}\right)}=\ln\frac{P\left(h_{1}\right)}{P\left(h_{2}\right)}+t\times \ln\frac{p\left(e|h_{1}\right)}{p\left(e|h_{2}\right)}\text{,}$$

which can be written in an analogous fashion for its counterpart $\ln\frac{P\left(h_{2}|e_{1..t}\right)}{P\left(h_{1}|e_{1..t}\right)}$ by interchanging $h_{1}$ and $h_{2}$. These log-odds are attractive candidates for computational models of neuronal processes of decision making because they (i) allow for optimal decision making and (ii) rise linearly over time, as shown in Fig. 2a. It is assumed that a hypothesis is accepted when its decision variable reaches a fixed threshold. This yields a decision rule that is evaluated at each point of time t:

(6) $$\mathrm{accept}\left\{\begin{array}{c} x_{k}=1\\ x_{k}=2 \end{array}\right\}\mathrm{when}\left\{\begin{array}{c} \ln\frac{P\left(h_{1}|e_{1..t}\right)}{P\left(h_{2}|e_{1..t}\right)}\\ \ln\frac{P\left(h_{2}|e_{1..t}\right)}{P\left(h_{1}|e_{1..t}\right)} \end{array}\right\}>\vartheta \text{,}$$

and otherwise neither is accepted. Note that equivalently optimal decision rules, although framed somewhat differently, have been proposed by previous authors. For example, in the case of a forced-choice task, Gold and Shadlen (2001) consider a decision rule that is based on the likelihood ratio of the two hypotheses: accept $x_{k}=1$ when $\frac{p\left(e_{1..t}|h_{1}\right)}{p\left(e_{1..t}|h_{2}\right)}>\frac{P\left(h_{2}\right)}{P\left(h_{1}\right)}$, and accept $x_{k}=2$ otherwise. This rule can be turned into a decision rule for our task by multiplying the right-hand side criterion by an additional factor c that introduces the necessary ‘temporal gap’ in which neither hypothesis is accepted. Equating ϑ≡lnc, taking logarithms, and using Eq. (1), this modified rule can be rewritten as

$$\frac{p\left(e_{1..t}|h_{1}\right)}{p\left(e_{1..t}|h_{2}\right)}>\frac{P\left(h_{2}\right)}{P\left(h_{1}\right)}\times e^{\vartheta }$$

$$\ln\frac{P\left(h_{1}\right)}{P\left(h_{2}\right)}+\ln\frac{p{\left(e|h_{1}\right)}^{t}}{p{\left(e|h_{2}\right)}^{t}}>\vartheta$$

(7) $$\ln\frac{P\left(h_{1}\right)}{P\left(h_{2}\right)}+t\times \ln\frac{p\left(e|h_{1}\right)}{p\left(e|h_{2}\right)}>\vartheta \text{,}$$

which is precisely the same rule as in (6). This means that the two approaches are decision-equivalent.

Both the decision variable for the true hypothesis in (7) and its counterpart for the alternative hypothesis start at specific initial levels that represent the subject’s prior, and then rise or fall, respectively, over time. This corresponds to the notion of the accumulation of supportive evidence for the two rival hypotheses. A saccade to the true target location is released at time τ when

$$\ln\frac{P\left(h_{x_{k}}|e_{1..\tau }\right)}{P\left(h_{{\bar{x}}_{k}}|e_{1..\tau }\right)}=\vartheta$$

$$\ln\frac{P\left(h_{x_{k}}\right)}{P\left(h_{{\bar{x}}_{k}}\right)}+\tau \times \ln\frac{p\left(e|h_{x_{k}}\right)}{p\left(e|h_{{\bar{x}}_{k}}\right)}=\vartheta$$

(8) $$\tau =\frac{\vartheta -\ln\frac{P\left(h_{x_{k}}\right)}{P\left(h_{{\bar{x}}_{k}}\right)}}{\ln\frac{p\left(e|h_{x_{k}}\right)}{p\left(e|h_{{\bar{x}}_{k}}\right)}}\text{,}$$

where $x_{k}$ and ${\bar{x}}_{k}\;\in \left\{1,2\right\}$ denote the true and the false target location of the current trial k, respectively.

The likelihood term $p\left(e|h_{i}\right)$ can be thought of as a descriptor of a subject’s visual discrimination efficiency or processing capacity. The larger it is the more quickly will an observed sensory stimulus make the subject increase their posterior belief in the corresponding hypothesis, and the shorter the resulting saccade latency. One way of parameterizing this quantity is to assume arbitrary ‘evidence units’. With

(9) $$p\left(e|h_{x_{k}}\right)=1+\rho \;\text{and}\;p\left(e|h_{{\bar{x}}_{k}}\right)=1$$

the supportive evidence per unit time for the true hypothesis is larger than the evidence for the false hypothesis, by an amount determined by a second model parameter ρ>0.

**Fig. 3:**
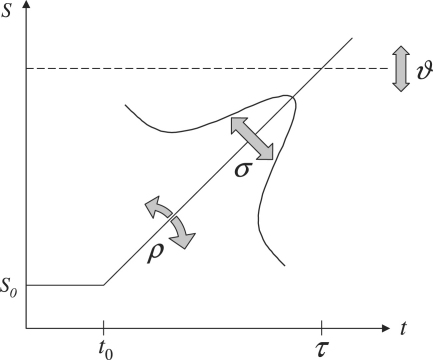
Intra-trial model parameterization. The proposed intra-trial model has three free parameters, represented by grey arrows. (i) ρ and (ii) σ determine the mean and the standard deviation of the normally distributed slope of the decision signal that corresponds to the true target location of the current trial. The larger ρ, the shorter the predicted saccade latency τ. The larger σ, the larger the variability of the distribution of τ. (iii) ϑ specifies the threshold the decision signal has to reach in order to evoke a saccade. The larger ϑ, the longer the latency and the less the influence of the initial value $S_{0}$.

In addition to the parameters ϑ and ρ, we must account for the fact that, across trials, the rate of the decision signal varies (see Fig. 2a). Previous experiments based on the same paradigm as in this study have found reciprocal latencies to conform to a Gaussian distribution (Carpenter & Williams, 1995). Hence, in trial k, the rate of the assumed decision signal, $r_{k}=\Delta S_{k}\frac{1}{\tau_{k}}$, will have a Gaussian distribution as well, with $\Delta S_{k}=S_{T}-S_{0,k}$ denoting the difference between the threshold $S_{T}$ and the initial level $S_{0,k}$ of the decision variable (see Fig. 2b). This introduces a third model parameter σ that describes the standard deviation of r. The parameterization of the intra-trial model is summarized in Fig. 3 .

#### 2.2.2 Inter-trial modelling

A central assumption of our model is that, at the beginning of each trial, the starting point of the decision signal associated with the true hypothesis corresponds to $\ln\frac{P\left(h_{x_{k}}\right)}{P\left(h_{{\bar{x}}_{k}}\right)}$, i.e. the log ratio of the prior probabilities of the correct and the incorrect hypothesis, see Eq. (5). The evolution of the prior probabilities $P\left(h_{i}\right)$ during a sequence of trials should reflect the learning of certain statistical properties of the target locations. In order to investigate what type of learning might happen during a sequence of trials $x_{1},x_{2},\ldots ,x_{N}$, we propose three different inter-trial models that formalize alternative learning hypotheses.

##### 2.2.2.1 The transition model

In the first candidate inter-trial model, we assume that subjects act like ideal observers: while responding to the sequence of stimuli within a block, they continuously refine an estimate of the underlying Markov transition matrix (see Minka (2001)).

Let $P=\left[p_{i,j}\right]$ be a hidden homogeneous transition matrix with a uniform initial distribution π=(0.5,0.5) underlying the sequence of target locations $x_{1},\ldots ,x_{N}$. The states of the Markov chain, 1 and 2, encode leftward and rightward targets, respectively. Let $n_{i,j}^{\left(k-1\right)}$ denote the number of transitions $x_{i}\to x_{j}$, for i,j∈{1,2}, that have occurred in the sequence of k−1 trials observed so far. A maximum likelihood estimate of P could be obtained by maximizing the log likelihood function

(10) $$\ln L\left(\overset{ˆ}{P};x_{1},\ldots ,x_{k-1}\right)=\underset{i,j}{\sum }\overset{\left(k-1\right)}{\underset{i,j}{n}}\ln{\overset{ˆ}{p}}_{i,j}$$

subject to $\sum_{j}p_{i,j}=1\;\forall i\in \left\{1,2\right\}$. Using Lagrange’s method, the maximum likelihood estimates ${\overset{ˆ}{p}}_{i,j}^{\mathrm{ML}}=\frac{n_{i,j}}{\sum_{l=1}^{2}n_{i,l}}$ follow.

For example, having observed the first six trials in Table 1, an ideal observer will have counted two ‘left→left’ transitions, one ‘left→right’ transition, and so forth. From this follows an estimated transition matrix ${\overset{ˆ}{P}}^{\mathrm{ML}}=\left[\begin{array}{cc} 0.67 & 0.33\\ 0.5 & 0.5 \end{array}\right]$. It is the matrix that makes the observed sequence of trials most likely.

In this form, however, an individual matrix element ${\overset{ˆ}{p}}_{i,j}^{\mathrm{ML}}$ remains undefined as long as $n_{i,1}+n_{i,2}=0$. Instead, we assume an initial uniform prior of $p_{i,j}^{\left(0\right)}=\frac{1}{2}$ for all i,j∈{1,2}, which can be thought of as an imaginary ‘prior observation count’ of 1 for each transition event (Minka, 2001). The posterior predictive distribution ${\overset{ˆ}{p}}_{i,j}^{\left(k\right)}$ based on k trials then allows subsequent generative models to yield predictions for all trials. Specifically, at the beginning of trial k, subjects are assumed to have constructed the estimate

(11) $${\overset{ˆ}{P}}^{\left(k-1\right)}={\left[{\overset{ˆ}{p}}_{i,j}^{\left(k-1\right)}\right]}_{i,j\in \left\{1,2\right\}}\;\mathrm{with}\;{\overset{ˆ}{p}}_{i,j}^{\left(k-1\right)}≔\frac{n_{i,j}^{\left(k-1\right)}+1}{\overset{2}{\underset{l=1}{\sum }}\left(n_{i,l}^{\left(k-1\right)}+1\right)}\text{,}$$

such that ${\overset{ˆ}{p}}_{i,j}^{\left(k-1\right)}>0\;\forall i,j,k$ and ${\overset{ˆ}{p}}_{i,j}^{\left(0\right)}=\frac{1}{2}\;\forall i,j$. Using this alternative formulation, the estimated transition matrix in the above example (Table 1) would be ${\overset{ˆ}{P}}^{\left(6\right)}=\left[\begin{array}{cc} 0.6 & 0.4\\ 0.5 & 0.5 \end{array}\right]$.

Obtaining maximum likelihood estimates of the transition matrix elements in this way can equivalently be viewed as a Bayesian update scheme. By counting how often each type of transition has occurred so far, an ideal observer can estimate the joint probabilities $P\left(x_{k-1}=i,x_{k}=j\right)$, from which estimates of the conditional probabilities ${\overset{ˆ}{p}}_{i,j}\equiv P\left(x_{k}=j|x_{k-1}=i\right)$ can be derived (see Harrison, Duggins, and Friston (2006), for an example).

The initial uniform matrix at the beginning of an experimental block corresponds to maximal uncertainty. As more and more trials are observed, the posterior of the transition matrix is refined and gradually approaches the true matrix.

##### 2.2.2.2 The state model

The inter-trial model outlined so far is *transition*-oriented in that it assumes an observer who estimates a transition matrix underlying the sequence of stimuli. Alternatively, a *state*-oriented observer can be imagined who simply estimates a state probabilities vector $\overset{ˆ}{p}=\left(p_{1},p_{2}\right)$ by counting the frequencies $n_{i}\;\forall i\in \left\{1,2\right\}$ of the two target locations while not paying attention to the transitions between them. At the beginning of trial k∈{1,…,N}, in analogy to the Bayesian update scheme outlined above, this estimate is

(12) $${\overset{ˆ}{p}}^{\left(k-1\right)}={\left[{\overset{ˆ}{p}}_{i}^{\left(k-1\right)}\right]}_{i\in \left\{1,2\right\}}\;\mathrm{with}\;{\overset{ˆ}{p}}_{i}^{\left(k-1\right)}≔\frac{n_{i}^{\left(k-1\right)}+1}{\overset{2}{\underset{l=1}{\sum }}\left(n_{l}^{\left(k-1\right)}+1\right)}\text{,}$$

such that ${\overset{ˆ}{p}}_{i}^{\left(k-1\right)}>0\;\forall i$ for all k∈{1,…,N}. Again, the initial prior is assumed to be uniform, ${\overset{ˆ}{p}}_{1}^{\left(0\right)}={\overset{ˆ}{p}}_{2}^{\left(0\right)}=\frac{1}{2}$.

Having observed the first six trials of the above example (Table 1), a ‘state’ observer will have counted four ‘left’ trials and two ‘right’ trials. Using (12), an estimated state probabilities vector ${\left(0.63,0.38\right)}^{T}$ follows. Using the same notation as in the transition model, this estimate can be written as ${\overset{ˆ}{P}}^{\left(6\right)}=\left[\begin{array}{cc} 0.63 & 0.38\\ 0.63 & 0.38 \end{array}\right]$.

##### 2.2.2.3 The uniform model

The two alternative inter-trial models proposed so far account for different forms of learning, but they do not question whether learning occurs at all. Therefore, a third candidate model is proposed in which subjects are assumed to be entirely ignorant of the history of stimuli. In this *uniform* model, subjects maintain a uniform prior belief of ${\overset{ˆ}{p}}_{1}={\overset{ˆ}{p}}_{2}=0.5$ in either target location throughout the experiment. Using the same notation as in the transition model, this prior can be written as a constant estimate ${\overset{ˆ}{P}}^{\left(k-1\right)}=\left[\begin{array}{cc} 0.5 & 0.5\\ 0.5 & 0.5 \end{array}\right]\;\forall k=1,\ldots ,N$.

**Fig. 4:**
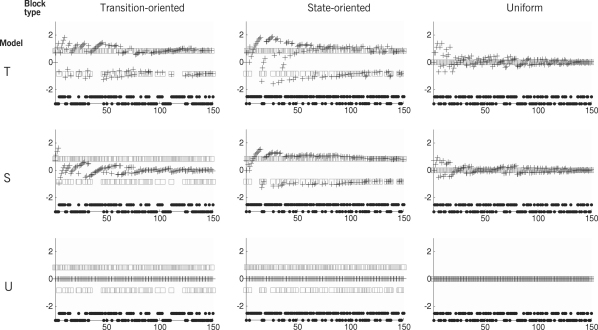
Log prior ratios predicted by the alternative inter-trial models. Each diagram is based on the combination of a particular block structure (transition-oriented, state-oriented, or uniform) and a particular inter-trial model (‘transition’ model, ‘state’ model, or ‘uniform’ model). For each trial, the diagrams show the target location (black dots), with high and low markers indicating leftward and rightward targets. Furthermore, they show the log ratio between the prior probability of the true and the false target location (grey squares) as well as a prediction for this log ratio, generated by the respective inter-trial model (black crosses). Since the models are always initialized with uniform priors, the predicted log ratio for trial k=1 is ln(0.5/0.5)=0. The upper left diagram, for example, shows how the ‘transition’ model gradually adapts to the transition-oriented block structure underlying the observed sequence of trials. By contrast, the central diagram in the left column shows that the ‘state’ model is incapable of learning the structure of a transition-oriented block.

Fig. 4 illustrates exemplary predictions generated by the alternative inter-trial models operating on alternative block structures. The individual diagrams show the extent to which the models are able to adapt to the transition matrix underlying the observed sequence of target locations. Crucially, the rate of convergence is highest when the model structure most closely matches the block structure. In particular, convergence takes longer when the true block structure is more complicated than the assumed one. For example, in the case of a uniform block, all three models eventually settle around 0. 5/0.5 predictions, but the ‘state’ model and the ‘transition’ model take longer to converge. Thus, we will be able to make use of measured reaction times from *all* blocks to find out which model explains a particular subject’s behaviour best (Section 3.4).

#### 2.2.3 Model construction

The two sub-models outlined above can now be integrated into an overall generative model with three free parameters.

In our paradigm, reciprocal latencies have previously been observed to follow a normal distribution (Carpenter & Williams, 1995). Thus, the rate of the rising decision signal can be modelled as a random variable

(13) $$R_{k}∼N\left(\mu_{r},\sigma^{2}\right)\text{,}$$

where $\mu_{r}$ and σ describe the mean and the standard deviation of the rate across trials. Using $\frac{1}{\tau }=\frac{r}{\Delta S}$, the reciprocal latency $y_{k}≔\frac{1}{\tau_{k}}$ can then be modelled as a random variable

(14) $$Y_{k}∼N\left(\frac{1}{\Delta S_{k}}\mu_{r},\frac{1}{{\left(\Delta S_{k}\right)}^{2}}\sigma^{2}\right)\text{.}$$

The difference $\Delta S_{k}$ between the initial level $S_{0,k}$ of the decision signal and its threshold can be expressed in terms of a model parameter ϑ and the log prior ratio as derived in Section 2.2.1:

(15) $$\Delta S_{k}=S_{T}-S_{0,k}=\vartheta -\ln\frac{P\left(h_{x_{k}}\right)}{P\left(h_{{\bar{x}}_{k}}\right)}\text{.}$$

The mean rate of the decision signal was derived in (5) and (9):

(16) $$\mu_{r}=\ln\frac{p\left(e|h_{x_{k}}\right)}{p\left(e|h_{{\bar{x}}_{k}}\right)}=\ln\left(1+\rho \right)\text{.}$$

The variability of the rate across trials is described by the model parameter σ. The prior of the two hypotheses is given by the corresponding entry in the transition matrix, as estimated within the inter-trial model:

(17) $$P\left(h_{x_{k}}\right)={\overset{ˆ}{p}}_{x_{k-1},x_{k}}\;\text{and}\;P\left(h_{{\bar{x}}_{k}}\right)={\overset{ˆ}{p}}_{x_{k-1},{\bar{x}}_{k}}=1-{\overset{ˆ}{p}}_{x_{k-1},x_{k}}\text{.}$$

The overall probability distribution for reciprocal latencies, conditional on the model parameters, is then given by

(18) $$Y_{k}|\rho ,\vartheta ,\sigma ∼N\left(\frac{\ln\left(1+\rho \right)}{\vartheta -\ln\frac{{\overset{ˆ}{p}}_{x_{k-1},x_{k}}}{1-{\overset{ˆ}{p}}_{x_{k-1},x_{k}}}},{\left[\frac{\sigma }{\vartheta -\ln\frac{{\overset{ˆ}{p}}_{x_{k-1},x_{k}}}{1-{\overset{ˆ}{p}}_{x_{k-1},x_{k}}}}\right]}^{2}\right)\text{.}$$

Note that the overall model does not propose a single parameterized distribution Y, but a sequence of distributions ${\left(Y_{k}\right)}_{1\leq k\leq N}$. This is because of its dependence on the output of the inter-trial model, ${\overset{ˆ}{p}}_{x_{k-1},x_{k}}$, which in turn depends on the sequence of target locations $\left(x_{1},\ldots ,x_{k-1}\right)$ observed so far.

### 2.3 Parameter estimation

Each candidate model constructed in the preceding section proposes a particular distribution of reciprocal saccade latencies parameterized by a vector θ=(ρ,ϑ,σ). Fig. 3 indicates that the parameters are not perfectly independent in their effect on the resulting predictions. For example, an increase in predicted response speed can be obtained by either decreasing the threshold ϑ or by increasing the rate of the decision signal ρ. Note, however, that this also alters the dependence of reaction times on the subject’s priors, so the parameters are not completely interchangeable. Nevertheless, to avoid numerical identifiability problems during parameter estimation, we reparameterized the model such that the rate ρ is expressed as per unit threshold, i.e. ρ→ρ/ϑ. Moreover, since the dispersion parameter σ must take a non-negative value, we used a log-transform. Thus, during numerical parameter estimation, the model was parameterized by $\theta^{\prime }=\left(\rho /\vartheta ,\vartheta ,\ln\sigma \right)$.

The maximum likelihood principle identifies those model parameters of the distributions of $Y_{1},\ldots ,Y_{N}$ that are most likely to give rise to the data $y_{1},\ldots ,y_{N}$. An estimate ${\overset{ˆ}{\theta }}_{\mathrm{ML}}=\left(\overset{ˆ}{\rho },\overset{ˆ}{\vartheta },\overset{ˆ}{\sigma }\right)$ can be found by maximizing

(19) $$\ln\overset{N}{\underset{k=1}{\prod }}p_{k}\left(y_{k}|\theta \right)=-\frac{N}{2}\ln2\pi -\overset{N}{\underset{k=1}{\sum }}\ln\frac{\sigma }{\vartheta -\ln\frac{{\overset{ˆ}{p}}_{x_{k-1},x_{k}}}{1-{\overset{ˆ}{p}}_{x_{k-1},x_{k}}}}-\overset{N}{\underset{k=1}{\sum }}\frac{{\left(y_{k}-\frac{\ln\left(1+\rho \right)}{\vartheta -\ln\frac{{\overset{ˆ}{p}}_{x_{k-1},x_{k}}}{1-{\overset{ˆ}{p}}_{x_{k-1},x_{k}}}}\right)}^{2}}{2{\left(\frac{\sigma }{\vartheta -\ln\frac{{\overset{ˆ}{p}}_{x_{k-1},x_{k}}}{1-{\overset{ˆ}{p}}_{x_{k-1},x_{k}}}}\right)}^{2}}$$

with respect to θ. The implementation can be simplified by omitting the term $-\frac{N}{2}\ln2\pi$ which does not depend on the free parameters. Multiplying the expression by (−1), the maximum likelihood estimate ${\overset{ˆ}{\theta }}_{\mathrm{ML}}$ is then found as the solution to a minimization problem with respect to ϑ, ρ, σ.

## 3 Results

### 3.1 Data analysis

**Fig. 5:**
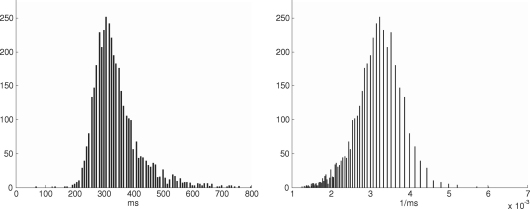
Histogram of latencies and reciprocals. The diagrams are based on 28 blocks containing 3 866 trials from subject S-1. While the latencies themselves have often been described as log normally distributed (Glimcher, 2003), their reciprocals can be approximated by a normal distribution (Carpenter & Williams, 1995).

In order to validate our paradigm, we replicated two key results by Carpenter and Williams (1995). First, latencies varied considerably over trials. Across our 20 078 trials, we found an overall mean of μ=262ms and a standard deviation of σ=65ms. Second, reciprocal latencies $\tau_{k}=1/y_{k}$, with $\mu =4.02\times 10^{-3}\;ms$ and $\sigma =1.67\times 10^{-3}\;ms$, closely approximated a normal distribution (see Fig. 5 ).

**Fig. 6:**
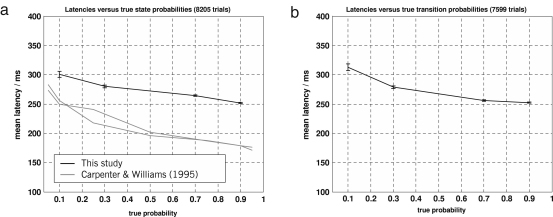
Saccade latencies and error bars. (a) Saccade latencies versus true state probabilities, based on all trials from state-oriented blocks across all subjects. (b) Saccade latencies versus true transition probabilities, based on all transition-oriented blocks across all subjects. Both diagrams show how saccade latencies decrease with increasing true probability of the respective target location.

Above and beyond these descriptive features, Carpenter and colleagues showed that latencies declined as the learned state probability of the corresponding target location increased (Basso & Wurtz, 1997, 1998; Carpenter & Williams, 1995). Specifically, they found a significant negative linear relation between log prior probabilities and latencies. In our study, we replicated these results as well, finding a very similar negative linear relation between log state probabilities and latencies in our state-oriented blocks (p<0.01). We further extended this analysis to transition probabilities. Using a linear regression analysis, we found a significant relation (p<0.01) between reciprocal latencies and log transition probabilities (see Fig. 6 ). Even though this analysis neglects the dynamics of learning completely, it already indicates that human observers are sensitive to transition probabilities in visual input statistics. Yet formal model comparison will provide much stronger evidence for this claim.

### 3.2 Parameter estimation

In order to obtain maximum likelihood parameter estimates from Eq. (19), a gradient-descent scheme was run on the acquired data from all three subjects separately. The results are given in Table 3 .

**Fig. 7:**
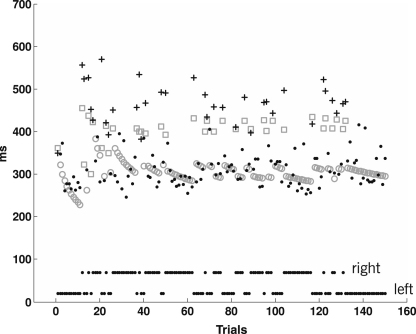
Averaged observed and predicted latencies. The diagram shows saccade latencies from an additional experimental session subject S-1 was engaged in. The dataset consists of 10 transition-oriented blocks, each designed to contain an identical left/right sequence of 150 target locations. The diagram shows the trial-by-trial target locations as separate lines of black dots at the bottom (leftward targets: lower line; rightward targets: upper line). The observed latencies, averaged over these 10 sessions, are plotted in black, and their respective model predictions in grey. Observations and predictions of trials in which the target location has stayed on the same side are shown as small black dots and grey circles, respectively. Observations and predictions of trials in which the target location has just switched to the other side are depicted as black crosses and grey squares, respectively. Predicted reaction times (grey circles and squares) show two key features of an ideal observer who is sensitive to transition probabilities. First, whenever there is a sequence of trials with identical target locations (a ‘run’), reaction times drop continuously as the estimated prior probability of that target location increases. Second, whenever the target changes to the other side (a ‘switch’), there is a single long-reaction-time trial, followed by a return to the previous, lower level of reaction times.

In order to visualize the dependence between conditional probabilities and latencies, and provide face validity for our parameter estimates, one of the subjects was engaged in an additional session consisting of 10 transition-oriented blocks with identical sequences initially generated from a $\left[\begin{array}{cc} 0.8 & 0.2\\ 0.2 & 0.8 \end{array}\right]$ transition matrix. We fitted the model to the data and predicted the priors ${\overset{ˆ}{p}}_{x_{k-1},x_{k}}$ using the ‘transition’ model. Fig. 7 shows how observed latencies develop over time and how this is reflected by model predictions.

**Fig. 8:**
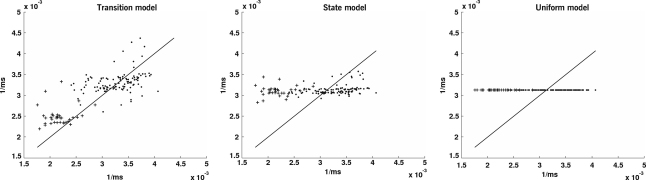
Averaged observed versus predicted reciprocal latencies from Fig. 7. In each of the diagrams, predicted reciprocal latencies (y-axis) are plotted against their observations (x-axis). The diagrams are based on the ‘transition,’ the ‘state,’ and the ‘uniform’ model, respectively, applied to the same dataset as in Fig. 7. Thus, the x-coordinates of the data points are the same in all three diagrams, whereas their y-coordinates differ. Predictions were generated by the alternative models after being individually fitted to the data. The main diagonal represents a perfect match between observations and predictions.

In order to give a qualitative comparison between the predictive power of the three competing inter-trial models, Fig. 8 visualizes measured latencies versus model predictions. The individual diagrams show a marked separation between trials in which the target has just switched to the other side (crosses) and those in which it has not (dots). As expected from a subject that learns transition probabilities, switch trials lead to long latencies, i.e. short reciprocals; accordingly, crosses have low x-coordinates. Conversely, non-switch trials lead to short latencies, i.e. long reciprocals; accordingly, dots have high x-coordinates. Looking at the y-coordinates, the ‘transition’ model is the only model that predicts these two clusters of trials.

### 3.3 Statistical classification

The parameter estimates obtained for each subject are sufficiently distinct to demonstrate the use of a statistical classifier that maps an unseen test sample onto the correct class (Jain, Duin, & Mao, 2000). Such a classifier could be used to (i) separate groups of individuals that exhibit similar learning profiles, or (ii) find out whether there are systematic differences between healthy subjects and patients.

A class can be regarded as a discrete random variable C and the eye movement data as a vector of continuous random variables $Y=\left(Y_{1},\ldots ,Y_{N}\right)$ with observed realizations $y=\left(y_{1},\ldots ,y_{N}\right)$. For illustration purposes, let the classes in $C=\left\{c_{1},c_{2},c_{3}\right\}$ correspond to the three subjects themselves. Then, given a test sample of unseen saccade data y, the classifier is to return

(20) $$c^{*}=\underset{c\in C}{\mathrm{argmax}}P\left(C=c|Y=y\right)\text{.}$$

Using Bayes’ theorem and the fact that saccade latencies $y_{1},\ldots ,y_{N}$ are independently drawn from their respective underlying distributions, the right-hand side can be rewritten as

(21) $$P\left(C=c|Y=y\right)=\frac{p\left(C=c,Y=y\right)}{p\left(y\right)}$$

(22) $$=\frac{P\left(C=c\right)\times p\left(y|C=c\right)}{p\left(y\right)}$$

(23) $$\propto P\left(C=c\right)\overset{N}{\underset{k=1}{\prod }}p_{k}\left(y_{k}|C=c\right)\text{.}$$

We based the classifier on the ‘transition’ model which showed a reasonable fit across all subjects, and trained it by finding maximum likelihood estimates of labelled training data from the three subjects (see Table 4 ). The unknown parameters of the distribution in Eq. (18) were then replaced by these estimates, yielding approximations for the class-conditional probability densities p(y|C=c). The priors were chosen to be flat, i.e. $P\left(C=c\right)=\frac{1}{3}\;\forall c\in C$. In future applications, when particular classes are to be distinguished such as subgroups of a disease, the priors would be given by the unconditional frequencies of the different conditions.

Table 5 shows the resulting joint probabilities p(c,y)∝p(c|y)∀c∈C as determined by the classifier.

The table shows that the classifier has assigned the test sample to the correct class in all cases.

Fig. 4 provides some intuition as to what specific properties of a sequence of latencies allow the classifier to distinguish between different inter-trial models. In a transition-oriented block (left column in the figure), for instance, the ‘transition’ model predicts that log prior ratios fall into two groups: negative and positive ones (top-left diagram). Accordingly, saccadic latencies are expected to be separated into long-latency and short-latency saccades. The ‘state’ model, by contrast, predicts the convergence of log prior ratios around zero (mid-left diagram), and saccadic latencies are expected to display relatively low variability around their mean. Finally, the ‘uniform’ model predicts that log prior ratios remain fixed at zero.

### 3.4 Model comparison

**Fig. 9:**
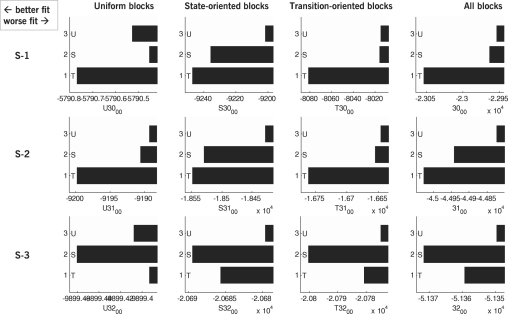
Negative log likelihood of the inter-trial models fitted to alternative subsets of the data. Each diagram shows the negative likelihood of the ‘uniform,’ the ‘state,’ and the ‘transition’ model when fitted to the data of a particular subject (rows) confronted with a sequence of target locations generated from a uniform, state-oriented, or transition-oriented block (columns). The rightmost column shows the negative likelihood of the model fitted to all blocks of a particular subject. The smaller the negative log likelihood, the better the model fit.

The maximum likelihood approach discussed so far does not only yield a point estimate of the model parameters. It can also be used to compare the competing inter-trial models. The question of which model explains the observed data best can be posed explicitly by comparing

(24) $$\ln p\left(y|M\right)={\langle \ln p\left(y,\theta |M\right)\rangle }_{q\left(\theta \right)}+{\langle \ln q\left(\theta \right)\rangle }_{q\left(\theta \right)}+D_{\mathrm{KL}}\left(q\left(\theta \right)|p\left(\theta |y,M\right)\right)\approx \ln\overset{N}{\underset{k=1}{\prod }}p\left(y_{k}|{\overset{ˆ}{\theta }}_{\mathrm{ML}}^{M},M\right)+\mathrm{const}$$

for each inter-trial model M, where we approximate the unknown parameter distribution q(θ) by a Dirac-delta distribution at ${\overset{ˆ}{\theta }}_{\mathrm{ML}}^{M}$. The likelihood term evaluated at the maximum likelihood parameter estimate, $p\left(y|{\overset{ˆ}{\theta }}_{\mathrm{ML}}^{M},M\right)$, is an approximation to the model evidence, p(y|M). When competing models do not differ with regard to their parameterization (as in our case, where model differences are restricted to the parameter-free function describing the ideal observer), these models can be compared using their likelihood ratio, which is an approximation to the model evidence ratio, or Bayes factor (see Kass and Raftery (1995)). Since the logarithm is a monotonic function, it is common practice, and usually more convenient, to compute and report the log likelihood ratio. Hence, differences between bar lengths in Fig. 9 represent the log likelihood ratio between the associated models (note that differences between logs are mathematically equivalent to the log of the ratio).

The negative log likelihood values of the competing inter-trial models fitted to different subsets of the data are given in Fig. 9. Each diagram is based on a particular subject and a particular type of block structure. Note that it is meaningless to compare absolute values between subjects since they depend on the mere number of trials a subject was engaged in. Instead, the likelihood value of each model must be interpreted in relation to the likelihood values of the other models from the same subject and block structure. Each diagram has been scaled individually so as to emphasize the full range between the lowest and the highest likelihood.

The data show that model fit differences vary considerably across subjects. In subjects S-1 and S-2, there is very strong evidence for the ‘transition’ model compared to the ‘uniform’ and the ‘state’ model. In subject S-1, for example, the likelihood ratio between the ‘transition’ model and the ‘state’ model, each fitted to all blocks, is $\exp\left(23\;053.79-22\;964.11\right)\approx 8.9\times 10^{38}$, that is, the ‘transition’ model is much more likely to underlie the observed data than the ‘state’ model. Similarly, it is $\exp\left(23\;053.79-22\;953.8\right)\approx 2.7\times 10^{43}$ times more likely than the ‘uniform’ model. In subject S-3, by contrast, the ‘state’ model allows for the best fit. For this subject, the likelihood ratios are much smaller than in the other two subjects, but they still constitute strong evidence in favour of the ‘state’ model. For the experimental data presented here, there was always strong evidence for one particular model in each combination of subject and block type.

The most salient result within the diagrams is that for each subject the same model is found to be optimal for all three data sets. The probability of this happening by chance is $\frac{3}{3^{3}}=\frac{1}{9}$ within each subject, and therefore $p={\left(\frac{1}{9}\right)}^{3}\approx 0.001$ for the three subjects as a whole.

## 4 Discussion

In this article, we have extended the class of linear rise-to-threshold models of the relation between a priori probabilities of target location and saccadic latencies. These models are centered around the intuitive, and empirically supported, notion of a decision signal that rises over time until reaching a fixed threshold. We have presented a generative, hierarchical model that combines two separate sub-models for learning of target locations across trials and the decision-making process within a trial, respectively. This has enabled three lines of progress: (i) explicit modelling of how subjects’ priors change across trials as a function of stimulus history, which makes it possible to investigate how saccadic decision making is dynamically shaped by learning; (ii) a model parameterization, inspired by computational considerations, which enables maximum likelihood parameter estimation and the subsequent construction of classifiers for distinguishing subjects with different learning profiles; (iii) differentiation between specific forms of learning by model comparison, e.g., the question of whether saccades are more influenced by marginal or by conditional probabilities.

The focus of this paper is a methodological one, and its primary goal is not to provide major novel neurobiological insights. Nevertheless, we acquired eye movement data from three healthy subjects performing a well-established and simple binary saccadic task. In order to make our results comparable to previous ones, we used the same paradigm as Carpenter and Williams (1995). The aim of this empirical data analysis was to demonstrate the face validity of our modelling approach by showing that under realistic noise levels, as found in standard saccade measurements with infrared video technology, consistent and subject-specific learning profiles can be identified. Indeed, our analysis showed that (i) the behavioural pattern observed in each subject was consistent across all experimental sessions and that (ii) it was distinct from the patterns of the other subjects.

The question that led to these findings was what sort of learning underlies changes of prior probabilities across trials in a particular subject. In our analyses of empirical data, we evaluated three different inter-trial learning models: a ‘state’ model (which learns the marginal probabilities of target locations), a ‘transition’ model (which learns the conditional probabilities of a transition matrix), and a ‘uniform’ model (representing the hypothesis that no learning takes place and prior probabilities therefore remain constant). Fitting the three alternative models to different subsets of the data shows that there is least evidence for the ‘uniform’ model in all cases. We can conclude from this that human observers do take into account the probabilistic structure underlying the sequence of trials. This finding was compatible with simple linear regression analyses of the relation between log prior probability ratios and reciprocal saccade latencies. In these analyses, we fully replicated the previous results by Carpenter and colleagues, who had reported a significant negative correlation between log prior marginal probabilities and reciprocal saccade latencies (Basso & Wurtz, 1997, 1998; Carpenter & Williams, 1995). In addition, we found a significant negative correlation between log prior transition probability ratios and reciprocal saccade latencies. This demonstrates that human observers are sensitive to conditional probabilities, not merely to the marginal probabilities, of target locations.

A particularly interesting finding is that in each of our three subjects there was one model (the ‘transition’ model in subjects S-1 and S-2, and the ‘state’ model in subject S-3) that performed best for all data sets, independent of the hidden probability structure underlying the stimulus sequence. The probability of obtaining such a pattern by chance is very small (p<0.01). In addition, model comparison, on the basis of likelihood ratios, showed that in each condition the best model allowed for a much better fit than both other models. This suggests that each subject exhibited an inherent and individual learning profile, independent of the current block type. This notion is also confirmed by our classification results, in which all unknown test samples were mapped onto the correct subject.

Although related modelling principles have been used in previous studies, these were implemented for rather different experimental paradigms. For example, Maddox (2002) has looked at a broad range of mathematical frameworks for optimal decision criterion learning in perceptual categorization tasks. In this type of experiment, subjects are asked to assign the correct category to a given stimulus, and a typical modelling approach is to assume that they aim to maximize expected reward. Smith, Wirth, Suzuki, and Brown (2007) consider a paradigm in which subjects are required to associate different cues with different actions. They propose a novel way of analysing such interleaved learning by means of a state-space model that provides a single framework for all associations to be learned. Corrado, Sugrue, Seung, and Newsome (2005) and Lau and Glimcher (2005) analyse sequential choice tasks with probabilistic reinforcers. They propose attractive candidate models that are capable of implementing the matching law described by Herrnstein (1961). Finally, several alternative models for two-choice reaction time tasks were compared by Ratcliff and Smith (2004), who showed that these models, even though based on very different assumptions, often led to similar predictions, at least for very simple tasks.

The above studies describe powerful approaches for characterizing and analysing learning and choice behaviour for a variety of tasks. In this study, by contrast, we were considering a different type of decision task, and we were aiming for a different type of insight. In our paradigm, subjects were not asked to assign a given sample to one of several overlapping categories; decisions were not associated with a reward; and there were no probabilistic reinforcers. Instead, subjects were confronted with a target that was extremely easily detectable, there were only two alternative responses per trial, and no behavioural feedback (e.g., rewards) was given. Similarly, we were not aiming to model the cognitive state of subjects who are evaluating the potential outcome of alternative decisions, or who are learning to associate a cue with a particular action. Instead, we attempted to model subjects’ reaction times in response to an extremely simple stimulus, and how these reaction times depended on learned statistical distributions about stimulus properties.

There are several potential lines of future research that could be based on the model presented in this paper. For example, an interesting extension would be to consider additional inter-trial models, e.g., differently parameterized ‘declining-memory’ models which represent forgetful observers, or observers that adapt their learning rate to the volatility of the environment (Behrens, Woolrich, Walton, & Rushworth, 2007). Because of different numbers of parameters in such models, however, model comparison could no longer be pursued on the basis of log likelihood ratios. Instead, one would require an approach that takes into account both model fit and model complexity, e.g., Bayesian model selection based on the model evidence (Penny, Stephan, Mechelli, & Friston, 2004). For example, different forms of online learning of the probability distribution from which a sequence of events is drawn have been used in the context of modelling SRTT neuroimaging data (Harrison et al., 2006; Strange, Duggins, Penny, Dolan, & Friston, 2005).

Another interesting question would be to investigate the extent to which the explanatory power of linear rise-to-threshold models is restricted to high-contrast settings in which the time taken for decision is often assumed to dominate the time for detection (Carpenter, 2004). This could be addressed by explicitly comparing linear-rise models to random-walk (or diffusion) models while systematically modifying the salience of the target (for a debate on the relationship between the two approaches see Carpenter and Reddi (2001) and Ratcliff (2001)).

Furthermore, the way the brain might implement Bayesian inference can be expressed in alternative mathematical ways (Jazayeri & Movshon, 2006; Ma, Beck, Latham, & Pouget, 2006; Rao, 2004). Current linear-rise models assume an implementation that is instantaneous in that the subject’s posterior belief in the competing hypotheses is calculated on the basis of evidence that evolves over time. That is, each piece of new evidence instantaneously leads to an update. Alternatively, Bayesian inference could be expressed in terms of a gradient-ascent scheme on the free energy which would converge to the true posterior probability of either hypothesis.

Given the dependence of learning on synaptic plasticity and neuromodulatory systems, the modelling approach described in this paper could be of interest for clinical applications, particularly in psychiatry (Stephan, Baldeweg, & Friston, 2006). One potential long-term target for clinical application of learning models of eye movements is schizophrenia. Among the most promising endophenotypes of schizophrenia are abnormalities both in antisaccade tasks (McDowell et al., 2002) and learning tasks (Stephan et al., 2006). These abnormalities are usually observed as subtle statistical discrepancies between groups of healthy subjects and patients. However, to our knowledge, these observations have not yet been successfully used for the development of richer classification systems as well as corresponding subject-specific diagnostics. Further investigation of the release of eye saccades and learning effects from a psychophysical perspective might help to detect systematic differences between healthy and diseased individuals in order to eventually improve early diagnosis. For example, classifiers (albeit more powerful ones than the example used in Section 3.3) could be used to map test samples from patients onto classes that correspond to different subgroups of a disease. In this way, Bayesian learning models could become a valuable tool for studying physiological and pathophysiological mechanisms of saccadic eye movements.

## Figures and Tables

**Box I:**
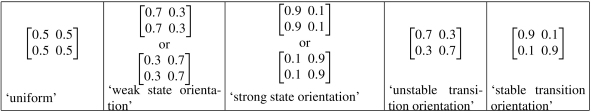


**Table 1:** Example of a sequence of target locations generated from the transition matrix $\left[\begin{array}{cc} 0.7 & 0.3\\ 0.3 & 0.7 \end{array}\right]$

Trial	1	2	3	4	5	6	…
Target probabilities	(0.5,0.5)	(0.7,0.3)	(0.7,0.3)	(0.7,0.3)	(0.3,0.7)	(0.3,0.7)	(0.7,0.3)
Target location drawn	$\begin{array}{c} \mathrm{Left}\;\left(1\right) \end{array}$	$\begin{array}{c} \mathrm{Left}\;\left(1\right) \end{array}$	$\begin{array}{c} \mathrm{Left}\;\left(1\right) \end{array}$	$\begin{array}{c} \mathrm{Right}\;\left(2\right) \end{array}$	$\begin{array}{c} \mathrm{Right}\;\left(2\right) \end{array}$	$\begin{array}{c} \mathrm{Left}\;\left(1\right) \end{array}$	…

On trial 1, the target location is always drawn from a uniform distribution (0.5,0.5). On all subsequent trials, its probability distribution depends on the target location of the previous trial.

**Table 2:** Number of blocks (B) and trials (T) with successfully extracted saccade latencies, per subject and type of transition matrix

Subject	Uniform		Weak state orientation		Strong state orientation		Unstable transition orientation		Stable transition orientation		All	
	B	T	B	T	B	T	B	T	B	T	B	T
S-1	7	948	7	985	4	557	5	691	5	685	28	3866
S-2	12	1727	12	1736	11	1554	11	1558	9	1285	55	7860
S-3	11	1599	11	1594	12	1779	12	1755	11	1625	57	8352

**Table 3:** Maximum likelihood parameter estimation for different inter-trial models (values rounded to 3 significant figures)

Inter-trial model	Subject S-1			Subject S-2			Subject S-3		
	ρ	ϑ	lnσ	ρ	ϑ	lnσ	ρ	ϑ	lnσ
Uniform model	0.0766	24.03	−4.18	0.0327	7.87	−5.06	0.0615	13.7	−4.95
State model	0.199	59.6	−3.28	0.126	29.6	−3.77	0.631	113	−2.85
Transition model	0.0724	23.5	−4.26	0.109	26.1	−3.92	1.37	200	−2.28

**Table 4:** Parameter estimation for subsequent classification, based on a subset of the original data (S-1: 6 out of 8 blocks. S-2: 9 out of 12 blocks. S-3: 9 out of 12 blocks)

Class	Training sample y	Parameter estimate (ρ,ϑ,lnσ)	P(C=c)
$c_{1}$	3049 out of 3866 trials	0.0724, 23.5, −4.26	0.333
$c_{2}$	5999 out of 7860 trials	0.116, 27.5, −3.89	0.333
$c_{3}$	6l35 out of 8352 trials	1.543, 216.32, −2.147	0.333

**Table 5:** Classification results

Subject	Test sample y	$\ln P_{30}$	$\ln P_{31}$	$\ln P_{32}$	$c^{*}$
S-1	817 trials	**0.495**×**10**^**4**^	0.395×10 ^4^	0.234×10 ^4^	$c_{1}$
S-2	1861 trials	0.873×10^4^	**1.05** ×**10**^**4**^	0.957×10 ^4^	$c_{2}$
S-3	2217 trials	0.880×10^4^	1.33×10 ^4^	**1.39** ×**10**^**4**^	$c_{3}$

The classifier was run on three test samples taken from the three subjects. It computed the joint probabilities p(c,y)∝p(c|y) for all classes $c_{1},c_{2},c_{3}$, and assigned each test sample to the class $c^{*}$ that maximized this joint probability (printed in **bold** font).

## References

[b1] Anderson B. (2008). Neglect as a disorder of prior probability. Neuropsychologia.

[b2] Asrress K.N., Carpenter R.H.S. (2001). Saccadic countermanding: a comparison of central and peripheral stop signals. Vision Research.

[b3] Basso M.A., Wurtz R.H. (1997). Modulation of neuronal activity by target uncertainty. Nature.

[b4] Basso M.A., Wurtz R.H. (1998). Modulation of neuronal activity in superior colliculus by changes in target probability. Journal of Neuroscience.

[b5] Behrens T.E.J., Woolrich M.W., Walton M.E., Rushworth M.F.S. (2007). Learning the value of information in an uncertain world. Nature Neuroscience.

[b6] Carpenter R.H.S. (2004). Contrast, probability, and saccadic latency; evidence for independence of detection and decision. Current Biology: CB.

[b7] Carpenter R.H.S., McDonald S.A. (2007). Later predicts saccade latency distributions in reading. Experimental Brain Research.

[b8] Carpenter R.H.S., Reddi B.A.J. (2001). Reply to ‘putting noise into neurophysiological models of simple decision making’. Nature Neuroscience.

[b9] Carpenter R.H.S., Williams M.L.L. (1995). Neural computation of log likelihood in control of saccadic eye movements. Nature.

[b10] Corrado G.S., Sugrue L.P., Seung H.S., Newsome W.T. (2005). Linear-nonlinear-poisson models of primate choice dynamics. Journal of the Experimental Analysis of Behavior.

[b11] Fischer B., Biscaldi M., Otto P. (1993). Saccadic eye movements of dyslexic adult subjects. Neuropsychologia.

[b12] Gitelman D.R. (2002). Ilab: A program for postexperimental eye movement analysis. Behavior Research Methods, Instruments, & Computers.

[b13] Glimcher P.W. (2001). Making choices: The neurophysiology of visual-saccadic decision making. Trends in Neurosciences.

[b14] Glimcher P.W. (2003). The neurobiology of visual-saccadic decision making. Annual Review of Neuroscience.

[b15] Gold J.I., Shadlen M.N. (2000). Representation of a perceptual decision in developing oculomotor commands. Nature.

[b16] Gold J.I., Shadlen M.N. (2001). Neural computations that underlie decisions about sensory stimuli. Trends in Cognitive Sciences.

[b17] Gold J.I., Shadlen M.N. (2002). Banburismus and the brain: Decoding the relationship between sensory stimuli, decisions, and reward. Neuron.

[b18] Grice G.R. (1968). Stimulus intensity and response evocation. Psychological Review.

[b19] Hanes D.P., Carpenter R.H.S. (1999). Countermanding saccades in humans. Vision Research.

[b20] Hanes D.P., Patterson W.F., Schall J.D. (1998). Role of frontal eye fields in countermanding saccades: Visual, movement, and fixation activity. Journal of Neurophysiology.

[b21] Hanes D.P., Schall J.D. (1996). Neural control of voluntary movement initiation. Science.

[b22] Hanes D.P., Thompson K.G., Schall J.D. (1995). Relationship of presaccadic activity in frontal eye field and supplementary eye field to saccade initiation in macaque: Poisson spike train analysis. Experimental Brain Research.

[b23] Harrison L.M., Duggins A., Friston K.J. (2006). Encoding uncertainty in the hippocampus. Neural Networks.

[b24] Herrnstein R.J. (1961). Relative and absolute strength of response as a function of frequency of reinforcement. Journal of the Experimental Analysis of Behavior.

[b25] Jain A.K., Duin R.P.W., Mao J. (2000). Statistical pattern recognition: A review. IEEE Transactions on Pattern Analysis and Machine Intelligence.

[b26] Jazayeri M., Movshon J.A. (2006). Optimal representation of sensory information by neural populations. Nature.

[b27] Kass R.E., Raftery A.E. (1995). Bayes factors. Journal of the American Statistical Association.

[b28] Kim J.N., Shadlen M.N. (1999). Neural correlates of a decision in the dorsolateral prefrontal cortex of the macaque. Nature Neuroscience.

[b29] Knill D.C., Pouget A. (2004). The Bayesian brain: The role of uncertainty in neural coding and computation. Trends in Neurosciences.

[b30] Kurata K., Aizawa H. (2004). Influences of motor instructions on the reaction times of saccadic eye movements. Neuroscience Research.

[b31] Lau B., Glimcher P.W. (2005). Dynamic response-by-response models of matching behavior in rhesus monkeys. Journal of the Experimental Analysis of Behavior.

[b32] Leach J.C., Carpenter R.H. (2001). Saccadic choice with asynchronous targets: Evidence for independent randomisation. Vision Research.

[b33] Loon E.M.V., Hooge I.T.C., Berg A.V.V.den (2002). The timing of sequences of saccades in visual search. Proceedings. Biological Sciences/The Royal Society.

[b34] Luce R.D. (1963). Detection and recognition.

[b35] Luce R.D. (1986). Response times: Their role in inferring elementary mental organization.

[b36] Ludwig C.J.H., Gilchrist I.D., McSorley E., Baddeley R.J. (2005). The temporal impulse response underlying saccadic decisions. Journal of Neuroscience.

[b37] Ma W.J., Beck J.M., Latham P.E., Pouget A. (2006). Bayesian inference with probabilistic population codes. Nature Neuroscience.

[b38] Maddox W.T. (2002). Toward a unified theory of decision criterion learning in perceptual categorization. Journal of the Experimental Analysis of Behavior.

[b39] Madelain L., Champrenaut L., Chauvin A. (2007). Control of sensorimotor variability by consequences. Journal of Neurophysiology.

[b40] McDowell J.E., Brown G.G., Paulus M., Martinez A., Stewart S.E., Dubowitz D.J. (2002). Neural correlates of refixation saccades and antisaccades in normal and schizophrenia subjects. Biological Psychiatry.

[b41] McMillen T., Holmes P. (2006). The dynamics of choice among multiple alternatives. Journal of Mathematical Psychology.

[b42] Minka T.P. (2001). Bayesian inference, entropy, and the multinomial distribution.

[b43] Nakahara H., Nakamura K., Hikosaka O. (2006). Extended later model can account for trial-by-trial variability of both pre- and post-processes. Neural Networks.

[b44] Nazir T.A., Jacobs A.M. (1991). The effects of target discriminability and retinal eccentricity on saccade latencies: An analysis in terms of variable-criterion theory. Psychological Research.

[b45] Newsome W.T. (1997). Deciding about motion: Linking perception to action. Journal of Comparative Physiology A.

[b46] Newsome W.T., Britten K.H., Movshon J.A. (1989). Neuronal correlates of a perceptual decision. Nature.

[b47] Newsome W.T., Britten K.H., Salzman C.D., Movshon J.A. (1990). Neuronal mechanisms of motion perception. Cold Spring Harbor Symposia on Quantitative Biology.

[b48] Newsome W.T., Pare E.B. (1988). A selective impairment of motion perception following lesions of the middle temporal visual area (mt). Journal of Neuroscience.

[b49] Oswal A., Ogden M., Carpenter R.H.S. (2007). The time course of stimulus expectation in a saccadic decision task. Journal of Neurophysiology.

[b50] Papoulis A. (1991). Probability, random variables, and stochastic processes.

[b51] Penny W.D., Stephan K.E., Mechelli A., Friston K.J. (2004). Comparing dynamic causal models. Neuroimage.

[b52] Platt M.L. (2002). Neural correlates of decisions. Current Opinion in Neurobiology.

[b53] Platt M.L., Glimcher P.W. (1999). Neural correlates of decision variables in parietal cortex. Nature.

[b54] Rao R.P.N. (2004). Bayesian computation in recurrent neural circuits. Neural Computation.

[b55] Ratcliff R. (1978). A theory of memory retrieval. Psychological Review.

[b56] Ratcliff R. (2001). Putting noise into neurophysiological models of simple decision making. Nature Neuroscience.

[b57] Ratcliff R., Cherian A., Segraves M. (2003). A comparison of macaque behavior and superior colliculus neuronal activity to predictions from models of two-choice decisions. Journal of Neurophysiology.

[b58] Ratcliff R., Rouder J.N. (1998). Modeling response times for two-choice decisions. Psychological Science.

[b59] Ratcliff R., Smith P.L. (2004). A comparison of sequential sampling models for two-choice reaction time. Psychological review.

[b60] Ratcliff R., Zandt T.van, McKoon G. (1999). Connectionist and diffusion models of reaction time. Psychological review.

[b61] Reddi B.A.J. (2001). Decision making: The two stages of neuronal judgement. Current Biology.

[b62] Reddi B.A.J., Asrress K.N., Carpenter R.H.S. (2003). Accuracy, information, and response time in a saccadic decision task. Journal of Neurophysiology.

[b63] Reddi B.A.J., Carpenter R.H.S. (2000). The influence of urgency on decision time. Nature Neuroscience.

[b64] Robinson D.A. (1973). Models of the saccadic eye movement control system. Biological Cybernetics.

[b65] Roitman J.D., Shadlen M.N. (2002). Response of neurons in the lateral intraparietal area during a combined visual discrimination reaction time task. Journal of Neuroscience.

[b66] Romaya, J. (2000). Cogent 2000. This experiment was realised using Cogent 2000 developed by the Cogent 2000 team at the FIL and the ICN and Cogent Graphics developed by John Romaya at the ION at the Wellcome Department of Imaging Neuroscience

[b67] Salzman C.D., Britten K.H., Newsome W.T. (1990). Cortical microstimulation influences perceptual judgements of motion direction. Nature.

[b68] Schall J.D. (2001). Neural basis of deciding, choosing and acting. Nature Reviews Neuroscience.

[b69] Schall J.D. (2003). Neural correlates of decision processes: neural and mental chronometry. Current Opinion in Neurobiology.

[b70] Schall J.D., Thompson K.G. (1999). Neural selection and control of visually guided eye movements. Annual Review of Neuroscience.

[b71] Shadlen M.N., Britten K.H., Newsome W.T., Movshon J.A. (1996). A computational analysis of the relationship between neuronal and behavioral responses to visual motion. Journal of Neuroscience.

[b72] Shadlen M.N., Gold J.I. (2004). The neurophysiology of decision-making as a window on cognition. The Cognitive Neurosciences.

[b73] Shadlen M.N., Newsome W.T. (1996). Motion perception: Seeing and deciding. Proceedings of the National Academy of Sciences of the United States of America.

[b74] Shadlen M.N., Newsome W.T. (2001). Neural basis of a perceptual decision in the parietal cortex (area LIP) of the rhesus monkey. Journal of Neurophysiology.

[b75] Sinha N., Brown J.T.G., Carpenter R.H.S. (2006). Task switching as a two-stage decision process. Journal of Neurophysiology.

[b76] Smith A.C., Wirth S., Suzuki W.A., Brown E.N. (2007). Bayesian analysis of interleaved learning and response bias in behavioral experiments. Journal of Neurophysiology.

[b77] Smith P.L., Ratcliff R. (2004). Psychology and neurobiology of simple decisions. Trends in Neurosciences.

[b78] Stephan K.E., Baldeweg T., Friston K.J. (2006). Synaptic plasticity and dysconnection in schizophrenia. Biological Psychiatry.

[b79] Sternberg S. (1969). The discovery of processing stages: Extensions of Donders’ method. Acta Psychologica.

[b80] Sternberg S. (1969). Memory scanning: Mental processes revealed by reaction-time experiments. American Science.

[b81] Strange B.A., Duggins A., Penny W., Dolan R.J., Friston K.J. (2005). Information theory, novelty and hippocampal responses: Unpredicted or unpredictable?. Neural Networks.

[b82] Thompson K.G., Bichot N.P., Schall J.D. (1997). Dissociation of target selection from saccade planning in macaque frontal eye field. Journal of Neurophysiology.

[b83] Thompson K.G., Hanes D.P., Bichot N.P., Schall J.D. (1996). Perceptual and motor processing stages identified in the activity of macaque frontal eye field neurons during visual search. Journal of Neurophysiology.

[b84] Usher M., McClelland J.L. (2001). The time course of perceptual choice: The leaky, competing accumulator model. Psychological Review.

[b85] Wald A. (1945). Sequential tests of statistical hypotheses. The Annals of Mathematical Statistics.

